# Reproducibility of automated opportunistic CT-based volumetric bone mineral density measurements at the thoracic spine

**DOI:** 10.1093/bjr/tqag175

**Published:** 2026-07-29

**Authors:** Philipp Raffler, Jannis Bodden, Katharina Schultes, Philipp Prucker, Daniela Pfeiffer, Arthur Wagner, Malek El Husseini, Jan S Kirschke, Sebastian Rühling

**Affiliations:** Department of Neuroradiology, TUM School of Medicine and Health, TUM Klinikum Rechts der Isar, Technical University of Munich, Munich, BY 81675, Germany; Department of Neuroradiology, TUM School of Medicine and Health, TUM Klinikum Rechts der Isar, Technical University of Munich, Munich, BY 81675, Germany; Department of Neuroradiology, TUM School of Medicine and Health, TUM Klinikum Rechts der Isar, Technical University of Munich, Munich, BY 81675, Germany; Department of Neuroradiology, TUM School of Medicine and Health, TUM Klinikum Rechts der Isar, Technical University of Munich, Munich, BY 81675, Germany; Department of Radiology, TUM School of Medicine and Health, TUM Klinikum Rechts der Isar, Technical University of Munich, Munich, BY 81675, Germany; Department of Neurosurgery, TUM School of Medicine and Health, TUM Klinikum Rechts der Isar, Technical University of Munich, Munich, BY 81675, Germany; Department of Neuroradiology, TUM School of Medicine and Health, TUM Klinikum Rechts der Isar, Technical University of Munich, Munich, BY 81675, Germany; Department of Neuroradiology, TUM School of Medicine and Health, TUM Klinikum Rechts der Isar, Technical University of Munich, Munich, BY 81675, Germany; TUM-Neuroimaging Center, TUM Klinikum Rechts der Isar, Technical University of Munich, Munich, BY 81675, Germany; Department of Neuroradiology, TUM School of Medicine and Health, TUM Klinikum Rechts der Isar, Technical University of Munich, Munich, BY 81675, Germany

**Keywords:** osteoporosis, automated vBMD, opportunistic screening, bone health assessment, reproducibility

## Abstract

**Objectives:**

Opportunistic CT-based screening for osteoporosis has been shown to be cost-effective and may help to reduce the existing treatment gap in patients. Reproducibility has not yet been investigated at thoracic levels, which is becoming increasingly relevant with upcoming lung cancer screening programs.

**Methods:**

Scans from 178 patients with 2 consecutive CT examinations within 1 month were retrospectively included. In all available reconstructions, automated extraction of trabecular volumetric bone mineral density (vBMD) was performed, including HU-to-BMD conversion and contrast phase correction. Mean trabecular vBMD was calculated for 2 thoracic regions (T5-T7 and T8-T10). Reproducibility was calculated as the root mean square coefficient of variation (RMSCV) under 6 reproducibility scenarios, ranging from different reconstructions of the same scan to measurements across different scanners and contrast phases.

**Results:**

In total, 914 vBMD observations were analyzed. RMSCV values ranged from 0.83% to 4.90% for T5-T7 and from 1.4% to 6.8% for T8-T10. One-way ANOVA showed a significant overall difference between reproducibility scenarios in both vertebral regions (all *P* < .001), with post hoc analyses demonstrating a stepwise increase in RMSCV with technical complexity.

**Conclusions:**

Our results support the clinical applicability of opportunistic osteoporosis screening at the thoracic spine across diverse CT acquisition protocols. With the upcoming national lung cancer screening programs utilizing low-dose CT (LDCT), an extraordinary opportunity emerges to address the substantial osteoporosis treatment gap through opportunistic bone health assessment.

**Advances in knowledge:**

This work is novel in extending reproducibility validation for opportunistic osteoporosis screening from the lumbar spine to thoracic vertebrae.

## Introduction

According to the SCOPE 2021 report, in 2019, approximately 32 million individuals in the EU27 + 2 were estimated to have osteoporosis, with a clear female predominance: while 22.1% of women aged ≥50 years were affected, the corresponding figure for men was 6.6%. In that same year, osteoporosis caused an estimated 4.3 million fragility fractures across the EU27 + 2 and was causally linked to approximately 248 000 deaths. The direct costs of osteoporotic fractures amounted to €56.9 billion, representing approximately 3.5% of total EU healthcare expenditure, yet only 3% of these costs were allocated to pharmacological treatment.^1^ Demographic trends are expected to further aggravate this burden: the annual number of fragility fractures is projected to rise by 24.8% between 2019 and 2034, from 4.3 to 5.3 million cases. Despite the availability of effective therapies, osteoporosis remains critically underdiagnosed and undertreated. The treatment gap among women aged ≥50 years at high fracture risk averages 71% across the EU27 + 2, primarily attributed to underdiagnosis and limited implementation of opportunistic screening strategies^1^^,^ ^2^ although they have been shown to be cost-effective.^3^^,^ ^4^

While dual-energy X-ray absorptiometry (DXA) remains the diagnostic gold standard, recent advances have enabled automated vertebral segmentation using convolutional neural networks, facilitating opportunistic osteoporosis screening from routine CT examinations without additional radiation exposure.^5–8^ The relevance of thoracic spine vBMD measurements has been demonstrated: Johannesdottir et al.^9^ found thoracic vertebral bone density strongly correlated with lumbar measurements and both regions reliably predicted incidental vertebral fractures, and Therkildsen et al.^10^ found that BMD measured from routine cardiac CT scans was significantly associated with fracture risk.

Upcoming nationwide lung cancer screening programs represent a significant opportunity for opportunistic bone mineralization assessment. Approved as a statutory insurance benefit in June 2025, the German program began in April 2026, providing annual low-dose computed tomography for high-risk individuals aged 50-75 years with a ≥ 25-year smoking history. Demographic analyses project approximately 5.5 million eligible individuals.^11^ The high volume of expected thoracic CT examinations presents an unprecedented opportunity for concurrent bone health assessment without additional radiation exposure or healthcare costs.

Previous work demonstrated reproducibility of opportunistic lumbar vBMD,^12^ but corresponding data for the thoracic spine are lacking. Therefore, the purpose of this study was to analyze reproducibility for automated thoracic vBMD measurements to support large-scale application within lung cancer screening programs.

## Methods

### Study population characteristics

Patients who underwent at least 2 consecutive routine thoracic or abdominal CT examinations between November 2016 and July 2020 were retrospectively identified from the institutional picture archiving and communication system (PACS). Eligible patients had an interscan interval of no more than 1 month and consistent regions of interest including at least 2 evaluable vertebrae. In total, 178 patients with 1326 observations, defined as 2 different acquisitions at the same vertebra level and patient, were included. Vertebrae deemed unmeasurable according to the ACR criteria^13^ were automatically excluded, specifically, fractured vertebrae or Modic type 3 changes affecting > 25% of the vertebral body. For quality assurance, 1 medical student and 2 neuroradiologists trained in spine imaging (K.S., 1 year of experience; J.B., 10 years of experience; J.S.K., 24 years of experience) reviewed the vertebral segmentations, automatic contrast phase detection, and evaluated the automated inclusion and exclusion of vertebrae, documenting any manual corrections, if necessary.

Numerous potential error sources exist in the CT-based bone mineral density analysis as described in-depth in Brunnquell et al.^14^ To account for this, subgroup definitions were adopted from Bodden et al.,^12^ where each group captures specific reproducibility scenarios, reflecting an aspect of technical or procedural variability in CT-based vBMD measurements:

1. Δrecon: both measurements obtained from the same acquisition, but with different reconstruction kernels or slice thicknesses
2. Δcontrast: measurements taken from different contrast phases within a single scan session
3. Δsession: measurements collected from different scan sessions using the same calibrated scanner during the same contrast media phase
4. Δscanner: measurements acquired during the same contrast media phase but at 2 separately calibrated scanners
5. Δall: measurements taken from 2 different scanners and during different contrast phases
6. scanner-agnostic: measurements obtained from 2 different scanners, with at least one scan performed on a non-asynchronously calibrated device, and only a tube voltage-specific calibration was utilized.

The patient cohort was divided into 2 groups. T5-T7 and T8-T10 were selected because of their localization in the central portion of the thorax. Upper thoracic vertebrae are frequently omitted or only partially covered, particularly in abdominal CT examinations, which limits their utility in opportunistic screening. The division into 2 separate groups allowed for a systematic assessment of potential level-dependent differences in reproducibility. Low-dose CT (LDCT) scans employed for lung cancer screening cover the entire thorax encompassing T5-T10, thus making these levels particularly relevant for screening applications. The vBMD measurements were performed by a fully automated, commercially available CE-marked framework based on several convolutional neural networks (SpineQ v. 1.1, Bonescreen GmbH).

### Data acquisition, scanner calibration, automated vertebral body segmentation, and vBMD extraction

In-house contrast-enhanced scans were acquired using a bodyweight-adapted dose (80 mL for ≤80 kg; 90 mL for 80-100 kg; 100 mL for >100 kg) of iodinated contrast agent (Imeron 300, Bracco Imaging Deutschland GmbH, Konstanz, Germany). The tube voltage was in the range between 80kVp and 130 kVp with an adaptive tube load. Image acquisition was conducted using 4 distinct in-house scanner models, while external imaging was performed across 15 different scanner types, as detailed in Table 1.

**Table 1:** List of in-house and external CT scanner models used for image acquisition in this study.

	Manufacturer	Scanner model
In-house	Siemens Healthineers, Erlangen, Germany	Somatom Definition AS+,
	Philips Medical Systems, Hamburg, Germany	IQon Spectral CT
		Brilliance iCT 256
		Ingenuity Flex
External	Canon Medical Systems, Amstelveen, Netherlands	Aquilion
		Aquilion PRIME
	Siemens Healthineers, Erlangen, Germany	Somatom Definition AS
		Somatom Force
		Somatom Emotion 6
		Somatom Emotion 16
		Somatom Sensation 40
	Philips Medical Systems, Hamburg, Germany	Biograph128
		Ingenuity Core 128
		Ingenuity CT
		Ingenuity Flex
		Precedence 16P
		Brilliance 40
	GE HealthCare Technologies, Chicago, United States	LightSpeed VCT
		Revolution EVO

We included all reformations with a spatial resolution of ≤3 mm craniocaudally and ≤5 mm anterior-posterior or left-right. The asynchronous calibration of in-house scanners was carried out using a commercially available anthropomorphic spine phantom (European Spine Phantom; Quality Assurance in Radiology and Medicine GmbH, Möhrendorf, Germany). In all reconstructions, spine detection, vertebral labeling, and trabecular compartment segmentation were fully automated using SpineQ (version 1.1 Bonescreen GmbH, Munich, Germany). Linear conversion factors based on scanner type and kVp were used to automatically perform HU-to-BMD calibration. A 2-dimensional DenseNET model was used for automated correction for the contrast media phase.^7^ Mean trabecular vBMD was calculated for each observation across the T5-T7 and T8-T10 thoracic vertebrae.

### Statistical analysis

All statistical calculations were performed using STATA 13.1 (StataCorp LLC, College Station, TX, United States) and MATLAB version R2024b (The MathWorks Inc., Natick, MA, United States). Absolute and relative interscan differences in vBMD (T5-T7) and vBMD (T8-T10) were calculated by subtracting the second from the first measurement and as a percent change, respectively. Median absolute and relative vBMD errors and their respective IQR were computed groupwise. Afterwards, root mean square coefficients of variation (RMSCV) were calculated for each group. Agreement between measurements was assessed using Bland-Altman plots, including 95% confidence intervals for the limits of agreement (mean difference ± 1.96 SD).

We determined statistical significance of group differences in absolute interscan vBMD differences across the 6 reproducibility scenarios via 1-way Analysis of Variance (ANOVA) followed by Tukey’s post hoc tests for pairwise comparisons.

Group differences between T5-T7 and T8-T10 were assessed separately for each reproducibility scenario using 2-sided independent-samples t-tests with Welch’s correction to account for unequal group sizes and variances.

## Results

After applying the exclusion criteria, this study included 178 patients contributing 914 observations. For the T5-T7 analysis, 122 patients provided 367 observations, while the T8-T10 cohort comprised 172 patients contributing 547 observations. The patient populations demonstrated similar demographic characteristics across both vertebral groups, with a slight male predominance in both cohorts (T5-T7: 62.3% male; T8-T10: 61.0% male).

The mean age of the whole cohort was 63.3 years. Within the different vertebral groups and reproducibility subgroups, the mean age was comparable and is shown in Tables 2 and 3. The Δrecon group provided the largest sample sizes in both vertebral regions (T5-T7: *n* = 150; T8-T10: *n* = 184). Conversely, the Δall group yielded the smallest sample sizes (T5-T7: *n* = 16; T8-T10: *n* = 32), as expected given the requirement for multiple varying parameters in the same patient. Most examinations were performed within the same institution, with out-of-house/in-house comparisons exclusively represented in the scanner-agnostic groups (T5-T7: *n* = 39; T8-T10: *n* = 57).

**Table 2:** Patient characteristics in the T5-T7 group.

Parameter	Unit	Δrecon	Δcontrast	Δsession	Δscanner	Δall	Scanner-agnostic
Patients	*n*	61	9	25	14	5	8
Observations	*n*	150	25	87	51	16	39
Sex^a^							
Male	*n* (%)	35	6	17	9	3	6
Female	*n* (%)	26	3	8	5	2	2
Age^a^ (years)	Mean ± SD	59.7 ± 14.7	65.2 ± 9.7	56 ± 14.8	58 ± 19.1	63.7 ± 10.1	61.1 ± 11.2
Location							
In-house/in-house	*n* (%)	145 (96.7%)	24 (96%)	87 (100%)	51 (100%)	16 (100%)	0
Out-of-house/in-house	*n* (%)	0	0	0	0	0	39 (100%)
Out-of-house/out-of-house	*n* (%)	5 (3.3%)	1 (4%)	0	0	0	0

a Values based on number of observations.

**Table 3:** Patient characteristics in the T8-T10 group.

Parameter	Unit	Δrecon	Δcontrast	Δsession	Δscanner	Δall	Scanner-agnostic
Patients	*n*	64	32	37	22	8	9
Observations	*n*	184	113	100	61	32	57
Sex^a^							
Male	*n* (%)	37	20	22	14	5	7
Female	*n* (%)	27	12	15	8	3	2
Age^a^ (years)	Mean ± SD	61.2 ± 14.3	63.8 ± 12.9	58.6 ± 15.6	59.9 ± 16.2	64.5 ± 9.3	59.8 ± 11.5
Location							
In-house/in-house	*n* (%)	173 (94.0%)	107 (94.7%)	100 (100%)	61 (100%)	32 (100%)	0
Out-of-house/in-house	*n* (%)	0	0	0	0	0	57 (100%)
Out-of-house/out-of-house	*n* (%)	11 (6.0%)	6 (5.3%)	0	0	0	0

a Values based on number of observations.

Figure 1 illustrates an exemplary patient who underwent 2 consecutive thoracic CT scans within a 3-day interval on the same scanner but with different contrast phases.

**Figure 1:**
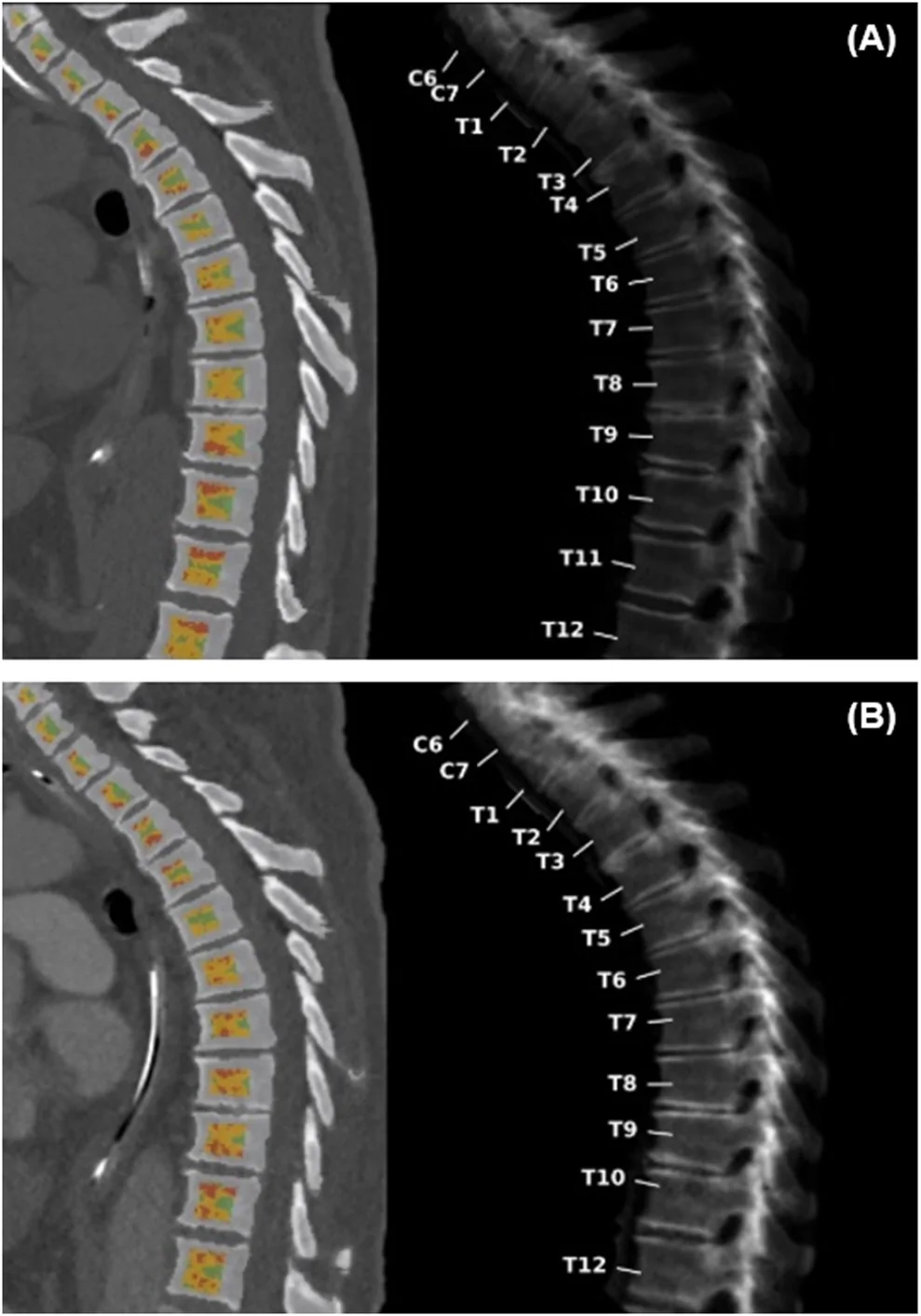
Thoracic CT examinations acquired with a 3-day interval on the same scanner. (A) Postoperative scan with oral contrast only; no intravenous contrast administered. The volumetric bone mineral density (vBMD) measured at T5-T7 was 111.5 mg CaHA/cm³. (B) Arterial-phase contrast-enhanced scan performed 3 days later. The vBMD measured at T5-T7 was 104.9 mg CaHA/cm³.

### T5-T7 reproducibility performance

Reproducibility values for the T5-T7 vertebral group are shown in Table 4 and demonstrate RMSCV ranging from 0.83% to 4.90%. Notably, the Δrecon group achieved the lowest RMSCV observed across all analyses. In the Δscanner group RMSCV was 4.90% (*n* = 51).

**Table 4:** Reproducibility of vBMD in T5-T7 according to each group.

Group name	Group size	Measurement 1 vBMD_T5-T7_ (mg CaHA/cm^3^)	Measurement 2 vBMD_T5-T7_ (mg CaHA/cm^3^)	Absolute difference (mg CaHA/cm^3^)	Relative difference (%)	RMSCV (%)

	*n*	Mean ± SD	Mean ± SD	Median (IQR)	Median (IQR)	
Δrecon*	150	200.0 ± 56.3	200.5 ± 56.6	1.8 (1.3-2.8)	1.0 (0.6-1.6)	0.8
Δcontrast*	25	176.2 ± 52.5	175.3 ± 55.6	5.2 (2.9-6.8)	3.5 (1.4-5.0)	2.3
Δsession*	87	202.8 ± 50.7	204.8 ± 56.5	10.8 (3.6-16.0)	4.9 (1.9-7.9)	3.5
Δscanner*	51	200.0 ± 68.6	202.3 ± 72.6	9.9 (5.8-11.7)	5.2 (2.5-8.2)	4.9
Δall*	16	194.6 ± 68.3	200.6 ± 68.0	10.8 (5.6-13.6)	4.2 (3.4-5.7)	2.7
Scanner-agnostic	39	211.6 ± 50.4	208.8 ± 44.8	10.9 (6.0-19.7)	4.9 (3.6-9.5)	3.4

Abbreviations: RMSCV, root mean square coefficients of variation; vBMD, volumetric bone mineral density. *asynchronously calibrated measurements only.

Bland-Altman plot analysis, shown in Figure 2, revealed mixed agreement for the subgroups. The number of measurements which exceeded the lower 95% CI of the upper limit of agreement (LoA) or the upper 95% CI of the lower LoA were 10 in the Δrecon subgroup (6.6%), 1 in Δcontrast (4.0%), 9 in Δsession (10.3%), 4 in Δscanner (7.8%), 2 in Δall (12.5%), and 1 in scanner-agnostic (2.6%).

**Figure 2:**
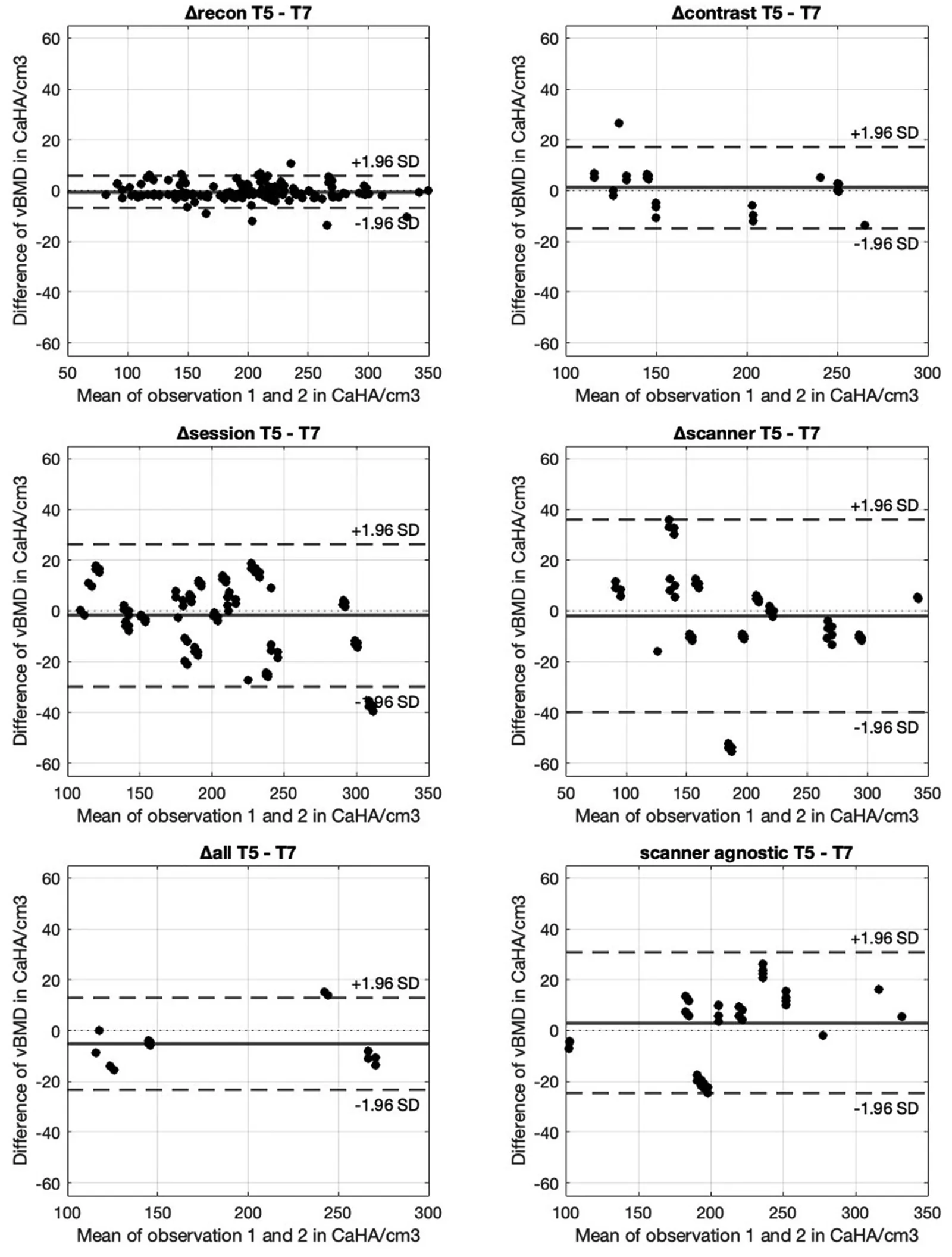
Bland-Altman Plots for the T5-T7 group. For each observation (dots), the difference between measurement 1 and 2 is plotted against the group mean which is indicated by the continuous black line. The long-dashed lines indicate the limits of agreement (±1.96 SD).

### T8-T10 reproducibility performance

Reproducibility values for the T8-T10 vertebral group are shown in Table 5. The Δrecon group achieved the lowest RMSCV in this group of 1.40%. The scanner-agnostic group achieved 4.70%. Δall demonstrated the highest RMSCV of 6.80% (*n* = 32) across the entire study.

**Table 5:** Reproducibility of vBMD in T8-T10 according to each group.

Group name	Group size	Measurement 1 vBMD_T8-T10_ (mg CaHA/cm^3^)	Measurement 2 vBMD_T8-T10_ (mg CaHA/cm^3^)	Absolute difference (mg CaHA/cm^3^)	Relative difference (%)	RMSCV (%)

	*n*	Mean ± SD	Mean ± SD	Median (IQR)	Median (IQR)	
Δrecon*	184	187.8 ± 52.3	187.2 ± 52.3	2.2 (1.2-3.8)	1.3 (0.6-2.4)	1.4
Δcontrast*	113	177.7 ± 54.4	173.2 ± 55.2	5.4 (3.0-9.8)	3.2 (1.7-5.5)	2.9
Δsession*	100	181.4 ± 53.6	185.3 ± 60.9	8.1 (3.5-16.3)	4.3 (1.8-10.3)	5.2
Δscanner*	61	192.3 ± 49.8	194.6 ± 52.0	8.2 (3.7-12.8)	4.2 (1.8-7.5)	4.9
Δall*	32	174.3 ± 43.9	179.0 ± 29.3	11.3 (6.0-20.6)	5.8 (3.7-9.9)	6.8
Scanner-agnostic	57	199.0 ± 44.9	191.7 ± 49.2	11.6 (7.5-19.5)	4.7 (3.2-11.2)	4.7

Abbreviations: RMSCV, root mean square coefficients of variation; vBMD, volumetric bone mineral density. *asynchronously calibrated measurements only.

In the Bland-Altman plot analysis shown in Figure 3, the number of measurements which exceeded the lower 95% CI of the upper LoA or the upper 95% CI of the lower LoA were somewhat smaller with 8 in the Δrecon subgroup (4.4%), 9 in Δcontrast (8.0%), 6 in Δsession (6.0%), 6 in Δscanner (9.8%), 2 in Δall (6.3%), and none in scanner-agnostic.

**Figure 3:**
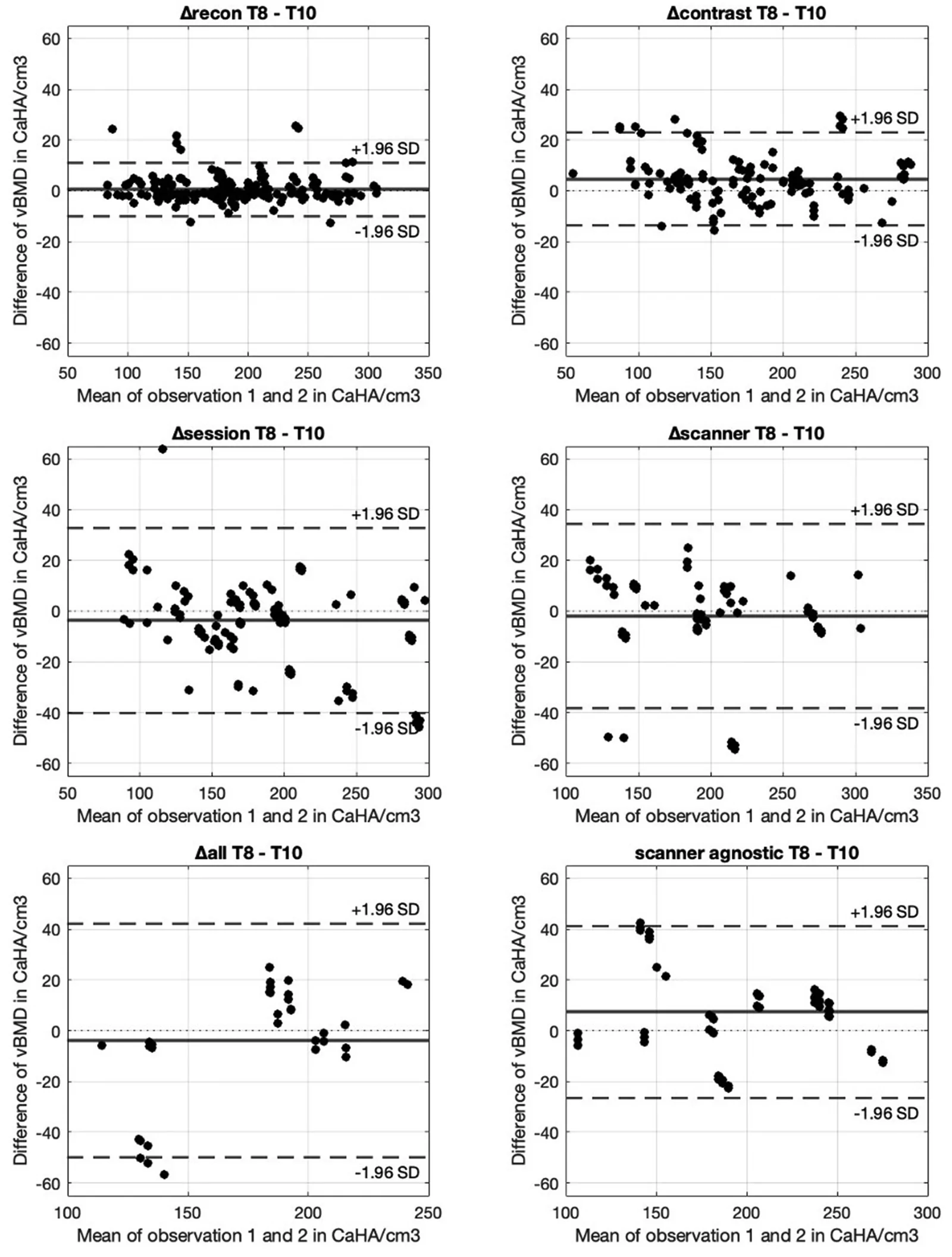
Bland-Altman Plots for the T8-T10 group. For each observation (dots), the difference between measurement 1 and 2 is plotted against the group mean which is indicated by the continuous black line. The long-dashed lines indicate the limits of agreement (±1.96 SD).

### Results of the ANOVA and Tukey post hoc tests

For T5-T7, 1-way ANOVA revealed a highly significant overall difference between the 6 reproducibility scenarios [F(5,361) = 27.9, *P* < .001]. Post hoc Tukey tests demonstrated significantly smaller absolute differences in Δrecon compared with Δsession, Δscanner, Δall and scanner-agnostic (all *P* ≤ .004), but not with Δcontrast (*P* = .21). Moreover, Δcontrast exhibited significantly lower absolute differences than Δscanner (*P* < .001), Δsession (*P* = .04) and scanner-agnostic (*P* = .01).

Similarly, for T8-T10, 1-way ANOVA demonstrated a highly significant overall difference across the 6 scenarios [F(5,541) = 23.6, *P* < .001]. Post hoc Tukey tests confirmed that Δrecon exhibited significantly smaller absolute differences than all other scenarios (all *P* ≤ .006). Additionally, Δcontrast showed significantly lower absolute differences than Δsession, Δall and scanner-agnostic (all *P* ≤ .003), but did not differ significantly from Δscanner (*P* = .06).

When comparing the reproducibility scenarios between the 2 vertebral level groups, absolute vBMD differences were significantly smaller in T5-T7 than in T8-T10 for both Δrecon (*P* = .005) and Δall (*P* = .02), reflected in lower RMSCV values of 0.83% vs. 1.4% and 2.7% vs. 6.8%, respectively. No significant differences were observed for the remaining reproducibility scenarios.

## Discussion

This study investigated the reproducibility of vBMD measurements of thoracic spine vertebrae across different technical conditions. The achieved RMSCV were comparable to existing literature for lumbar measurements.

The measurements for Δrecon demonstrated low RMSCV in both thoracic regions (T5-T7: 0.83%; T8-T10: 1.4%), comparable to prior work,^12^ highlighting the robustness of the automated framework against variations in reconstruction parameters. The parameter Δrecon can be regarded as analogous to interobserver variability, since both assess the consistency of repeated measurements derived from the same data. In Mueller et al.,^15^ interobserver variability for internally calibrated BMD was 4%, whereas Engelke et al.^16^ reported reproducibility errors of 1%-1.5% for trabecular bone and 2.5%-3% for cortical bone in spine measurements in 29 postmenopausal women.

Vertebral BMD is known to increase after contrast enhancement, which may lead to a clinically meaningful overestimation of vBMD and thus false-negative classifications, underscoring the necessity of phase-specific correction.^17–19^

Prior to deep learning-based automated correction, the standard approach to contrast phase adjustment relied on phase-dependent linear correction equations derived from triphasic CT acquisitions. Kaesmacher et al. systematically compared synchronous and asynchronous phantom calibration with phantomless internal tissue calibration across non-enhanced, arterial, and portal-venous phases, demonstrating that linear regression-based correction could reduce systematic bias for all 3 methods. However, reproducibility errors remained substantial, particularly for internal calibration in the arterial phase (RMSCV 8.8%).^20^

Ruhling et al.^7^ showed that the automatic phase correction using a 2D DenseNET achieved an accuracy of 98.3% and outperformed other artificial neural network algorithms. Using this model in our study, Δcontrast demonstrated RMSCV values < 3% comparing favorably with the reproducibility errors of 3.1%-8.8% reported by Kaesmacher et al. who used linear correction equations without dedicated automated phase correction.

Cross-session measurements (Δsession) displayed moderately higher RMSCV which aligns with expected challenges related to patient repositioning and temporal physiological variations. Baum et al.^21^ reported a notably higher RMSCV of 7.7% for 2 consecutive abdominal CT scans within 8 weeks and in Mueller et al.,^15^ longitudinal variability was 5.3%. This suggests improved precision in the current protocol. Patient-related factors such as positioning are likely to affect vBMD measurements.^22^ Additionally, QCT studies in specific populations suggest that changes in body composition, particularly visceral adipose tissue, are independently associated with lumbar vBMD.^23^

Cross-scanner variability has been described in literature: Zhao et al.^24^ found considerable variations in the vBMD between different scanners despite using the same phantom. Carpenter et al. analyzed the inter-scanner variability in QCT measurements of the proximal femur using 6 anthropomorphic standardization phantoms on 2 different CT scanners, reporting uncorrected inter-scanner coefficients of variation ranging from 3.8% to 15.7%. No phantom-based correction could decrease inter-scanner variability to intra-scanner precision.^25^ In the present study, Δscanner showed comparable performance in both thoracic levels that agreed well with previous literature.^12^

The scanner-agnostic approach yielded RMSCV values of 3.4% (T5-T7) and 4.7% (T8-T10), which are comparable to previously reported lumbar measurements.^12^ In thoracoabdominal CT of patients with chronic kidney disease, Therkildsen et al.^26^ reported errors of 5.8% for internally and 1.8% for asynchronously calibrated BMD estimates, while Wang et al.^27^ found intra-observer variability of approximately 2%-3% at lumbar levels L1-3 in asynchronously calibrated CT images. Collectively, these findings and the present RMSCV support the feasibility of simplified implementation strategies in multi-center environments, in which calibration can be performed at the level of scanner models or scanner types rather than requiring dedicated calibration protocols for each individual scanner unit.

As expected, the Δall group, representing the most challenging scenario with combined scanner and contrast phase variations, showed the overall highest RMSCV, consistent with prior work.^12^

The ANOVA and Tukey post hoc analyses further quantified the demonstrated hierarchy of precision across technical scenarios. For both thoracic regions, Δrecon showed significantly smaller absolute vBMD differences than all more complex scenarios (with the exception of Δcontrast in T5-T7). Absolute differences increased significantly from Δcontrast to Δsession and Δall, confirming that the degree of technical complexity has a measurable impact on reproducibility. These results emphasize that standardized acquisition and reconstruction protocols are essential to limit variability in vBMD measurements as highlighted by Brunnquell et al.^14^

The significantly smaller absolute vBMD differences in Δrecon and Δall for T5-T7 compared to T8-T10, reflected in lower RMSCV may stem from anatomical differences between the 2 regions. Vertebrae in the lower thoracic region are adjacent to the diaphragmatic domes and therefore may be more susceptible to respiratory motion artifacts.^28^

To contextualize the RMSCV clinically, we used the Least Significant Change (LSC) concept recommended by the International Society for Clinical Densitometry (ISCD), defined as LSC = 2.77 × RMSCV. It represents the minimum change between 2 measurements required to conclude with 95% confidence that an observed difference reflects true biological change rather than measurement error.^29^^,^ ^30^ In the present study, LSC values ranged from 2.3% (Δrecon, T5-T7) to 18.8% (Δall, T8-T10). Given that age-related vertebral trabecular bone loss proceeds at approximately 1.6% per year in women and 0.8% per year in men,^31^ the LSC of 2.3% under standardized single-scanner conditions corresponds to approximately 1-3 years of expected physiological bone loss, confirming that true vBMD change can be reliably detected within a clinically meaningful follow-up interval. In contrast, an LSC of 18.8% in the most challenging reproducibility scenario would require at least one decade of bone loss to exceed the 95% confidence interval for measurement error. This precludes reliable longitudinal monitoring and emphasizes the necessity of standardized single-center acquisition conditions in vBMD follow-up.

Opportunistic bone health assessment offers a promising screening strategy for early detection of osteoporosis and fracture risk stratification. A recent meta-analysis by Praveen et al.^32^ demonstrated that CT-derived biomarkers such as vBMD provide significantly higher discriminative performance for fracture prediction compared to conventional areal BMD measurements. This highlights the clinical relevance of accurate and reproducible vBMD measurements for opportunistic osteoporosis screening.

Some limitations need to be addressed. First, the retrospective study design limits control over scanning protocols and patient factors, potentially introducing unmeasured confounders. Second, the 1-month interval, while necessary to exclude biological vBMD changes, might represent a potential selection bias: Patients requiring 2 CT scans within 1 month are likely to have more severe underlying conditions, which may introduce physiological confounders beyond technical measurement variability. Third, the automated segmentation and contrast phase correction pipelines used in this study were originally developed and optimized at the same institution, partly on overlapping scanner types. Although both have been validated on external datasets, this may still introduce a subtle single-center bias and could overestimate reproducibility compared with fully independent multi-center settings and therefore may limit generalizability.

## Conclusion

The demonstrated reproducibility extends the previous validation from lumbar vertebrae to thoracic levels and supports the clinical applicability of opportunistic screening across diverse CT acquisition protocols. The hierarchy in reproducibility across different technical scenarios suggests that standardized acquisition parameters are essential for minimizing reproducibility errors especially in longitudinal vBMD assessment. Clinical confounders such as acute fluid shifts (eg, pleural effusions) should be excluded to ensure reliable detection of bone density changes over time.

With the impending implementation of national lung cancer screening programs utilizing LDCT, an extraordinary opportunity emerges to address the substantial osteoporosis treatment gap through opportunistic bone health assessment. Establishing reliable thoracic spine vBMD measurement protocols could enable simultaneous evaluation of both lung cancer and osteoporosis risk.
